# Sleep and physical activity: results from a long-term actigraphy study in adolescents

**DOI:** 10.1186/s12889-022-13657-0

**Published:** 2022-07-12

**Authors:** Chiara E. G. Castiglione-Fontanellaz, Tammy T. Timmers, Stefan Lerch, Christoph Hamann, Michael Kaess, Leila Tarokh

**Affiliations:** 1 University Hospital of Child and Adolescent Psychiatry and Psychotherapy, University of Bern, Bolligenstrasse 111, Haus A, 3000 Bern, Switzerland; 2 Translational Research Center, University Hospital of Psychiatry and Psychotherapy, University of Bern, Bern, Switzerland; 3 Department of Child and Adolescent Psychiatry and Psychosomatic Medicine, University Children’s Hospital, Inselspital, Bern University Hospital, University of Bern, Bern, Switzerland; 4 Department of Child and Adolescent Psychiatry, Center for Psychosocial Medicine, University Hospital Heidelberg, Heidelberg, Germany

**Keywords:** Adolescence, Sleep, Physical activity, Actigraphy

## Abstract

**Purpose:**

Research to date suggests that physical activity is associated with improved sleep, but studies have predominantly relied on self-report measures and have not accounted for school day/free day variability. To address these gaps in the literature, the aim of the present study was to (a) quantify physical activity in adolescents using long-term daily actigraphy measurement and (b) to examine the association between actigraphically assessed steps and sleep behavior in a sample of healthy adolescents. To be able to capture intra- and inter-individual differences in the daily physical activity of adolescents, we examined within as well as between subjects effects and its association with sleep.

**Methods:**

Fifty adolescents between 10 and 14 years of age were included in the present study. In total 5989 days of actigraphy measurement (average of 119 ± 40 days per participant; range = 39–195 days) were analyzed. We use multilevel modeling to disentangle the within and between subject effects of physical activity on sleep. In this way, we examine within an individual, the association between steps during the day and subsequent sleep on a day-to-day basis. On the other hand, our between subjects’ analysis allows us to ascertain whether individuals with more overall physical activity have better sleep.

**Results:**

Within a subject more steps on school and free days were associated with later bed times on school and free days as well as later rise times on school days only. On the other hand, comparing between subjects’ effects, more steps were associated with lower sleep efficiency on free and school days. No other significant associations were found for the other sleep variables.

**Conclusion:**

Our results obtained through objective and long-term measurement of both sleep and number of steps suggest weak or non-significant associations between these measures for most sleep variables. We emphasize the importance of the methodology and the separation of within subject from between subject features when examining the relationship between physical activity and sleep.

## Background

Children making the transition to adolescence experience marked changes in sleep behavior. The most striking of these changes is a trend towards later bedtimes, which has consistently been shown worldwide in this age group [1]. This delay in bedtime is driven by biological changes to the circadian timing system [2, 3] which favors later bedtimes in adolescence and is further exacerbated by environmental and psychosocial factors, such as homework, after-school activities, autonomy from parents, socialization and the use of technology (e. g., [4, 5]). Despite going to bed later, on school days adolescents wake up as early or even earlier than they did during mid and late childhood due to school start times [6], resulting in an overall decrease of total sleep duration and making them one of the most sleep deprived age groups [7]. Many adolescents attempt to make up for the sleep deficit accumulated during the school week with longer sleep on weekends [1]. This trend of inadequate and ill-timed sleep represents a major public health concern (e. g [8, 9]). There is ample evidence that insufficient sleep negatively impacts many domains of a teenager’s life, including cognitive functioning [10–13], academic performance [7, 14] and mental health [15–17]. Therefore, it is critical to identify factors that may facilitate healthier sleep in this population; one such factor may be physical activity [18–20].

The idea that a physically demanding day will lead to a good night’s sleep has existed since Biblical times “Sweet is the sleep of a laboring man…” (Ecclesiastes 5:11 as cited in [21]. Recent and large epidemiological surveys consistently show that regular physical activity is believed to be the most important sleep promoting behavior by the general public [22, 23] and many sleep experts consider it a non-pharmacological and cost-effective sleep aid [24, 25]. Despite the assumption that exercise has a beneficial effect on sleep, the empirical evidence supporting this assertion is inconclusive [25, 26]. A number of observational and experimental studies have found greater physical activity to be associated with less daytime sleepiness (measured subjectively) [27] earlier bedtimes (measured subjectively and objectively) [28], shortened latency to fall asleep (measured subjectively and objectively) and fewer awakenings at night (measured subjectively and objectively) [29–31]. Most recently, a multinational study of 5779 children aged 9–11 years found that moderate-to-vigorous intensity physical activity measured with a waist-worn actigraph was associated with longer sleep duration measured with the same device. However the effect sizes in this study were small [32].

In contrast, opposite and null effects of exercise on sleep have also been reported for children, adolescents and adults [33–35]. For example, Youngstedt and colleagues conducted two prospective studies examining the association between physical activity and sleep in young and older adults [36]. In the first study, 31 college students kept a diary for 105 consecutive days documenting their total exercise duration and a host of sleep variables including measures of sleep duration and quality. In the second study of older adults, 71 participants wore an actigraphy to measure physical activity and reported on their sleep using sleep diaries for seven consecutive days. In both studies, no noteworthy correlations were found between physical activity and sleep. Two further studies examining daytime physical activity and sleep in pre-adolescents (age 6–10 years) using actigraphy for seven consecutive days indicated that more physical activity was associated with more frequently interrupted sleep [37] and decreased sleep duration and sleep efficiency [38] on the following night.

From a methodological point of view, the mixed results from the studies described above may be attributed to different modes of measuring physical activity and sleep, which ranged from self-report questionnaires (often only comprised of one or two questions [39]) to objective measures via actigraphy and polysomnography. Interestingly, a systematic review of physical activity and sleep reported that of 21 studies only two studies relied exclusively upon objective measures [39]. Although self-report measures are often used due to their feasibility and cost-effectiveness, they have been shown to be inaccurate in younger populations. Adolescents tend to overestimate their physical activity levels, especially their vigorous physical activity [30] and may in particular report the most recent, salient and/or socially desirable patterns of sleep [39]. In a meta-analytic review, acute exercise was found to have limited beneficial effects on measures of sleep (e.g. total sleep time, sleep onset latency and sleep efficiency). On the other hand, regular exercise was found to have a small positive influence on total sleep time and sleep efficiency, a small to medium positive influence on sleep onset latency and a moderate positive influence on sleep quality [18].

The duration for which physical activity and sleep are measured may further muddle results. While appropriate measurement periods are device dependent, most studies examined exercise and sleep for only one or two days [36]. Prior research suggests that in order to achieve a reliability of 0.80 in adolescents, 8–9 days and 6–7 nights are required for valid actigraphy measured physical activity and sleep outcomes, respectively [40, 41]. Furthermore, if the measurement period is less than one week, care must be taken to include both school and free day activities given the large differences in sleep [42] and physical activity [43] on free as compared to school days [41].

Therefore, the primary aim of the present study was to overcome methodological limitations in previous studies by investigating the association between physical activity and sleep using long-term (i.e., several months) objective measurement of both factors in adolescents on both school and free days. We examine separately the associations between these parameters within a subject (steps during the day influencing subsequent sleep on a day-to-day basis) and between subjects (inter-individual variability in steps and its association with sleep). A secondary aim of the study was to report on normative values of number of steps for age, sex and school versus free day in early adolescents. Based on results from earlier research, we hypothesize that physical activity will differ on school as compared to free days [43]. With regards to sleep, results for this data set have previously been published [44], and as expected based on the existing literature sleep was shorter on school days as compared to free days and sleep duration declined with increasing age. Finally, despite inconsistent findings in the literature, we hypothesize that higher actigraphy-assessed physical activity in adolescents will be associated with better objective sleep.

## Methods

### Participants

Participants were recruited through flyers, advertisements and direct mailings to schools in the German speaking part of Switzerland as part of a twin study examining the heritability of sleep neurophysiology and behavior [44–49]. Exclusion criteria included suffering from a chronic or current illness, use of medications affecting sleep and brain function, known sleep disorders, and preterm birth before the 30th gestation week. The ethics commission of the canton of Zurich approved the study and participants and their parents provided written informed consent.

Participants with a minimum of 30 days of activity and sleep data were included in the analyses; eleven participants (7 girls, 4 boys) were excluded from the analyses because they did not meet this criteria. Therefore, the analysis is based on 50 participants with complete data (24 girls, 26 boys aged 12.78, ± 1.02 years). Mean body mass index was in the healthy range for adolescents (mean = 17.77; range = 13.88–22.03; *SD* = 1.95). Pubertal status was assessed with the Self-Rating Scale for Pubertal Development [50]. Of all participants 7 were prepubertal (3 females, 4 males), 14 individuals were early pubertal (14 males), 15 were midpubertal (9 females, 6 males), 12 were late pubertal (10 females, 2 males) and 2 were postpubertal (2 females).

### Procedures

Steps were assessed objectively and non-invasively with the Jawbone triaxial accelerometer (Jawbone, San Francisco, CA, USA) which participants wore on their non-dominant wrist. Participants were instructed to wear the Jawbone at all times for six months except while bathing or swimming. The Jawbone is considered an accurate and reliable device for monitoring physical activity [51], showing a test-retest reliability as revealed through an intra-class coefficient (ICC) of 0.97 for step count and 0.60 for active time [52]. Given the lower test-retest reliability of active time, we use number of step count for further analysis. The activity charts of every measurement day were visually inspected and data was excluded from the analyses if sequences during the waking hours indicated two or more consecutive hours of idle time, suggesting that the monitor had been removed from the body. Thus, in total 5989 days of data were available for analysis. The average number of school days, defined from Monday thru Friday, available for analysis was 66.86 ± 22.59 days (range: 22–108 days) per participant while the number of free days, meaning Saturday and Sunday (defined as weekends) and holidays ranged between 16 and 93 days with a mean of 52.92 ± 19.95 days per participant. The same approach was used for the sleep variables: Monday to Friday were counted as school days and Saturday as well as Sunday were defined as weekends. Holidays were defined as free days. We note that we use steps as a proxy for physical activity and thus use the terms physical activity and steps interchangeably.

The same activity monitor (Jawbone) was also used to assess sleep behavior across the 6-month interval. This device has also been validated for the measurement of sleep in adolescents [53]. Participants were instructed to press a button on the wristband before bed in the evening and upon waking in the morning to switch the device from the “active” to the “sleep” mode. If participants did not press the button to activate the sleep mode or active mode, the data was not included in the analysis. Using proprietary algorithms Jawbone calculates the following variables with minute precision for each night: Bed time, wake time, total sleep time (TST; time from sleep onset to sleep offset), sleep onset latency (SOL; time between going to bed and falling asleep), wake after sleep onset (WASO; time spent awake after sleep onset), number of awakenings (NOA) and sleep efficiency (SE; ratio of sleep time to time in bed). We note that this algorithm has previously been validated against polysomnography and shows good sensitivity and accuracy [54]. De Zambotti and colleagues report good agreement between Jawbone and polysomnography for TST (overestimated on average by 10.0 ± 20.5 min), SE (overestimated on average by 1.9% ± 4.2%), SOL (no difference), and WASO (underestimated by 9.3 min ± 20.4 min) in healthy adolescents (*n* = 65; mean age = 15.8 ± 2.5 years) [53]. Because sleep efficiency is a composite measure taking SOL, NOA, and WASO into account we use SE as our outcome variable along with TST and bed and rise times. As with steps, analyses were performed separately for school and free days.

For the present study the association between number of steps during the day and sleep on the subsequent night both measured via actigraphy was examined.

### Statistical analyses

 Mixed models for the four sleep outcomes (i.e., SE, TST, bed and rise times) were calculated to examine the effect of number of steps on sleep. Our predictor, physical activity described as number of steps per day was split into within and between subject measures. To perform between subject analyses, the mean number of steps across all measurement days was calculated for each individual (STEPS_M) resulting in one value per participant. In order to examine whether within an individual, days with more steps were associated with more sleep on the subsequent night, we adjusted for baseline levels of steps by subtracting the mean across all days for an individual by the number of steps per day (STEPS_D). 

Two-level mixed-effects regression analyses with robust variance estimation and standard errors were used to investigate the effect of age, gender, day type (school day versus free day) and number of steps on sleep. For each sleep outcome a separate mixed effect model is estimated. Data are grouped by subject, where we allow for a random intercept. To account for the different activity levels on school and free days, we included the interaction term DAYTYPE x STEPS_M (between subject analysis) and DAYTYPE x STEPS_D (within subject analysis), respectively. We use Cohen’s *f*^*2*^ to determine effect sizes [55]. Statistical significance was set to α = 0.05. All analyses were conducted in Stata/SE 16.1 [56].

In order to assess the impact of gender, age, and day type on steps, we performed a linear mixed model with random intercept grouped by subjects. For the analysis, age was centered by the sample mean (12.78 years) and the interaction of gender and day type was taken into account.

## Results

Results examining the impact of demographic variables and day type on steps are reported in Table 1.

**Table 1:** Results of the model examining the impact of age, gender, and day type (school versus free day) on number of steps. Fixed factors are shown and steps are measured in units of 1000 steps. Gender, Day Type, and Age were significant predictors of steps. Cohen’s f^2^ is used as a measure of effect size. All effect sizes are small

Factor	Coefficient	Cohens f^**2**^	z	P	95% CI
Gender (Girl)	−1.65	< 0.001	−2.57	0.01	−2.92, −0.40
Day Type (Free Day)	−1.63	0.013	−3.71	0.00	−2.49, −0.76
Interaction (Girl x Free Day)	−0.27	< 0.001	−0.45	0.65	−1.43, 0.89
Age	−0.80	< 0.001	−3.36	0.00	−1.26, −0.33
Intercept	23.35	< 0.001	7.58	0.00	17.32, 29.39

Table 2 summarizes the mean number of steps and sleep variables over all study participants.

**Table 2:** Mean values for age, number of steps averaged within a participant and subsequently averaged across participants separately for boys and girls. Bedtime and rise time are reported in clock time, total sleep time in hours and sleep efficiency defined as a percentage of total sleep time divided by time in bed

Factor	Mean	Standard deviation	Mean Girls	Standard deviation Girls	Mean Boys	Standard deviation Boys
Age (years)	12.78	1.01	12.66	1.24	12.88	0.77
Steps	11,608	2087	10,685	4759	12,242	5717
Bedtime (h)	21:17	1.0	20:51	0.53	21:41	1.17
Risetime (h)	06:56	0.91	06:37	0.32	07:15	1.16
Total Sleep Time (h)	8.87	0.93	8.98	0.61	8.77	1.16
Sleep Efficiency (%)	91.97	4.78	92.03	5.29	91.91	4.37

Averaging across days and participants, 11,609 steps were taken daily. We note, that the within subject variability (i.e., standard deviation) in the number of steps (4918 steps) is about 2.4 times higher as compared to the between subject variability (2087 steps) suggesting that while some adolescents were on average more active than others, there is a high day-to-day variation in the number of steps of an individual. This is also reflected in the intraclass correlation coefficient (ICC) for steps which was 0.10 (CI; 0.08, 0.14). Averaging across days and participants, the mean bedtime was at 21:17 o’clock, mean total sleep time was  8.87 hours and mean sleep efficiency was 91.97% . Mean bedtime in girls was at 20:51 o’clock, compared to 21:41 o’clock in boys. Total sleep time was comparable with girls sleeping 8.98 hours and boys sleeping 8.77 hours. Sleep efficiency was also similar (92.03% in girls, 91.91% in boys).

**Fig. 1:**
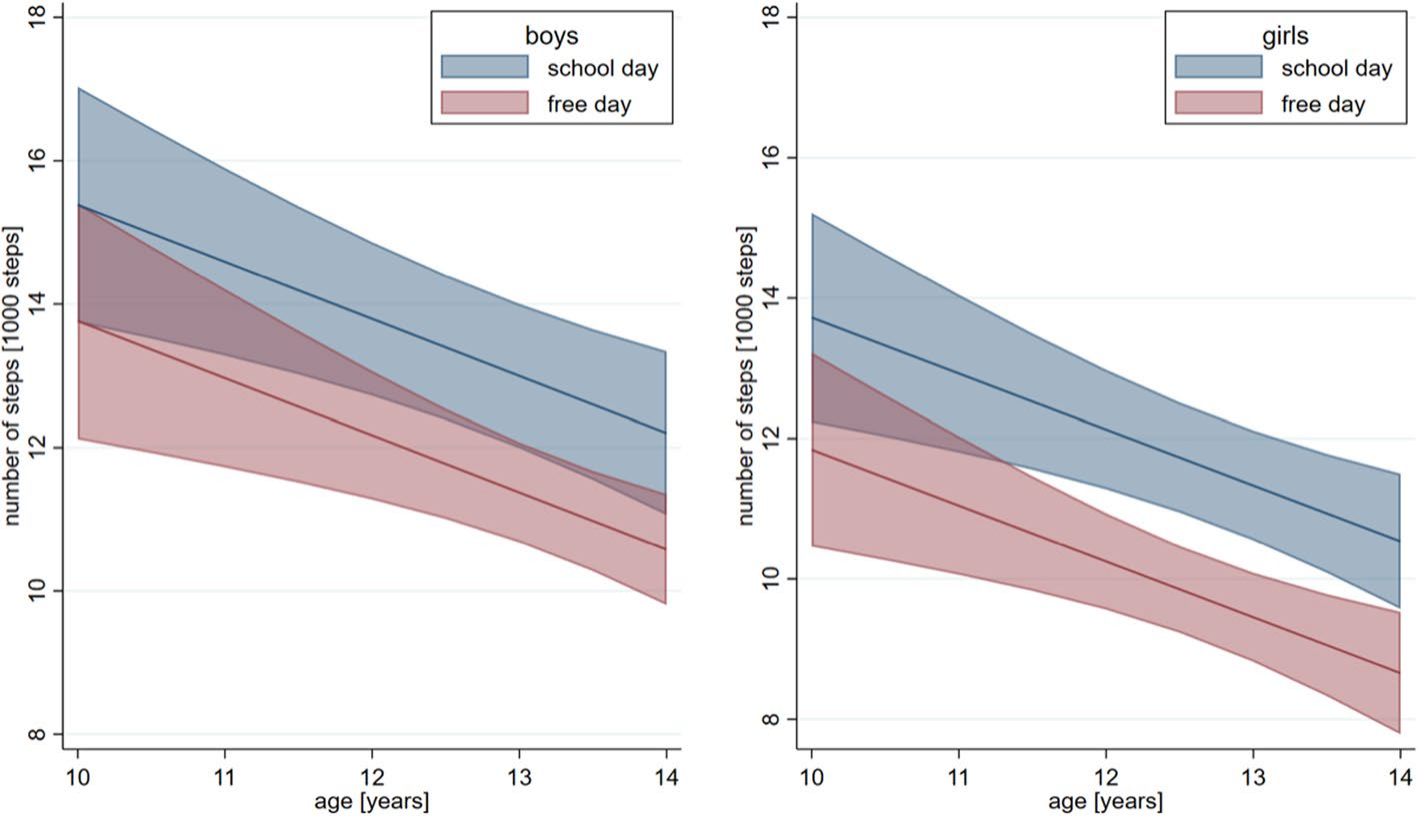
Predicted number of steps. Number of steps (in 1000 steps) is shown on the y-axis while age (in years) is on the x-axis. On the left side we see the predicted activity for boys and on the right side for girls (in blue for school days, in red for free days)

Boys took significantly more steps than girls, with girls taking on average 1650 steps less than boys (*p* = 0.01; Fig. 1). Both boys and girls were significantly more active on school days as compared to free days, with an average of 1630 (1900 for girls) fewer steps taken on free as compared to school days (*p* < 0.001, Fig. 1). Furthermore, a significant decline in the number of steps was observed with age (*p* < 0.001), with each year accounting for 800 fewer steps (*p* < 0.001).

Results showing the association between step count and sleep are presented in Table 3. Within subjects analyses revealed that taking more steps on school and free days was associated with later bedtimes (z = 2.37, *p* < 0.05, f^2^ = 0.00, free days; z = 6.43, *p* < 0.001, f^2^ = 0.01, school days). In other words, within an individual taking 1000 more steps was associated with a bedtime that was 1 minute later. Moreover, more steps were associated with later rise times the following morning on school (z = 3.10, *p* < 0.01, f^2^ = 0.00), but not free (z = 1.76, *p* = 0.08, f^2^ = 0.00) days. On school days, taking 1000 steps more was associated with a rise time that was 1.07 minutes later. No within subject associations were found between steps and total sleep time and sleep efficiency (see Table 3).

**Table 3:** Results of model with fixed factor and subject as random effect. In this table findings are divided between within and between subjects effects and free and school days. In this table, TST = total sleep time; SE = sleep efficiency. Data is reported in thousand steps (ksteps)

Factor	Coefficient	Cohens f^**2**^	z	p	95% CI
**Bedtime (in minutes)**					
Steps (between subjects; free day)	0.92	1.844e-06	0.37	0.70	−3.88, 5.71
Steps (between subjects; school day)	−1.95	9.418e-06	−1.05	0.29	−5.60, 1.70
Steps (within subjects; free day)	1.00	0.004	2.37	0.02	0.18, 1.84
Steps (within subjects; school day)	1.89	0.010	6.43	0.00	1.32, 2.50
**Rise time (in minutes)**					
Steps (between subjects; free day)	0.75	9.837e-07	0.34	0.73	−3.63, 5.13
Steps (between subjects; school day)	−1.70	< 0.001	− 0.88	0.38	−5.50, 2.08
Steps (within subjects; free day)	0.62	0.001	1.76	0.08	−0.07, 1.31
Steps (within subjects; school day)	1.07	0.002	3.10	0.00	0.39, 1.75
**TST (in minutes)**					
Steps (between subjects; free day)	−2.64	< 0.001	−1.63	0.10	−5.81, 0.53
Steps (between subjects; school day)	−2.03	< 0.001	−1.25	0.21	−5.19, 1.14
Steps (within subjects; free day)	−0.07	< 0.001	− 0.27	0.79	− 0.61, 0.46
Steps (within subjects; school day)	−0.47	< 0.001	−1.37	0.17	−1.13, 0.20
$\overset{\sim }{\boldsymbol{SE}}$ **(in percent)**					
Steps (between subjects; free day)	−0.44	< 0.001	−2.31	0.02	−0.82, − 0.07
Steps (between subjects; school day)	−0.43	< 0.001	− 2.55	0.01	− 0.75, − 0.10
Steps (within subjects; free -day)	0.04	< 0.001	1.61	0.11	−0.01, 0.10
Steps (within subjects; school day)	0.04	< 0.001	1.30	0.19	−0.2, 0.11

Between subjects, we find that people that took more steps on average have lower sleep efficiency on both free and school days (z = 2.31, *p* < 0.05, f^2^ = 0.00 free days; z = 2.55, *p* = 0.01, f^2^ = 0.00, school days). Here, taking 1000 steps more was associated with a reduction in sleep efficiency of about 0.44%. No other between subjects’ effects were observed.

## Discussion

The primary aim of the present study was to investigate the relationship between objectively-measured (i.e., actigraphy) daytime physical activity and sleep. Overall, our results demonstrate that physical activity declined during adolescence, that boys were more active than girls, and both boys and girls were more active on school as compared to free days. When looking at the association between actigraphy-assessed physical activity and sleep, we find that adolescents with more activity on school days, as indexed by mean steps per day, had slightly later bed and rise times as well as somewhat lower sleep efficiency.

As hypothesized, and in line with previous studies [43, 57, 58] we find that adolescents were less active on free days compared to school days. It is plausible that adolescents are more active during school days due to greater opportunities to participate in physical education classes and extra-curricular organized physical activity. This may be due to formalized physical activity policies at schools, which have been shown to have an influence on children’s activity levels [59] or teens may be prone to more sedentary behavior (i.e., computer use and television viewing) on free days [57, 58]. Thus, it is not only vital to implement physical activity policies in schools, but it is also essential to promote physical activity on free days.

Furthermore, as expected, we found that male adolescents were more physically active than their female counterparts, a finding that is consistent with previous studies examining physical activity of boys and girls with actigraphy (e.g., [19]). Previous research points to several possible explanations as to why girls are less physically active than boys. For instance, although schools provide opportunities to be physically active during school breaks and physical education class, they might be more readily accessible or desirable to boys. Indeed, in a survey study girls often reported that sports in high school are competitive, which is more likely to be enjoyed by boys [20].

Finally, as hypothesized and in agreement with previous studies conducted in several other countries [60], we found a significant decline in physical activity with increasing age. As this pattern is consistent across settings, it suggests that the decline may be normative during adolescence due to competing interests and additional academic pressure, which may reduce the time available for physical activity [61]. Taken together, these findings demonstrate how social and cultural factors can play a crucial role in the distribution of physical activity. Hence, the consistency of findings across studies highlight the need to consider the unique activity patterns of adolescents and factors influencing physical activity when developing physical activity intervention programs for this population.

Our second hypothesis that higher physical activity would be associated with better sleep quantity and quality was not confirmed. Our findings were inconsistent with some previous epidemiological, experimental and observational studies reporting a beneficial effect of physical activity on sleep [25, 30, 31]. Participants with more steps showed slightly lower sleep efficiency. This was an unexpected finding but in line with a cohort study examining the relationship between actigraphy-assessed physical activity and sleep in 275 Finnish pre-adolescents [38]. Their findings indicated that higher physical activity during the day was associated with shorter sleep duration, lower sleep efficiency and higher fragmentation of sleep the following night. Another explanation for the negative association between physical activity and sleep might be found in a trait-like inter-individual variability in activity level. For example, the inherent activity level of a child, might manifest itself in a higher activity level during both the day and the night [38]. A meta-analysis containing of 16 studies comparing sleep in children with attention-deficit/hyperactivity disorder (ADHD) versus controls, showed that children with ADHD had more bedtime resistance, sleep onset difficulties and night awakenings [62]. Therefore, it is suggested that the “trait” activity level may not only explain physical activity levels during the day but may also define the level of activity during the night [38].

Our within subject finding of more physical activity being associated with later bedtimes may be explained within the context of the current sleep hygiene recommendations that advise against exercise in the evening because of the negative impact on sleep [63]. In a meta-analysis higher exercise intensity was associated with prolonged sleep onset latency, lower total sleep time, lower sleep efficiency and more wake after sleep onset, if exercise ended around one hour prior to bedtime [63]. The somewhat detrimental effect of physical activity on sleep may be interpreted through the impact of physical activity on the circadian system. Physical activity can act as a “Zeitgeber” on the circadian system and has been shown to influence the phase of the circadian clock [64, 65]. Future studies should examine not only the association between physical activity and sleep, but also the timing of physical activity. Finally we would like to emphasize that we observe a one-minute delay per thousand steps. This small shift in sleep timing is unlikely to have meaningful consequences for sleep, however, a reduction of 1000 steps per day has measurable consequences for physical and mental health [66, 67]. Therefore, while our results help elucidate the association between physical activity and sleep, we caution against the use of these data to endorse less physical activity to promote better sleep.

The findings in this study need to be interpreted in light of some caveats and limitations. For example, we note that while the impact of physical activity on sleep was statistically significant and robust, the effect sizes are small and the benefits of physical activity likely outweigh the detrimental effects of such activity on sleep. Furthermore, even though actigraphy is an objective measure, there are still some limitations to it. First, the actigraphy used in the current study measured the overall physical activity and was not able to distinguish between different intensity levels. This may be key in explaining the mixed findings. Preliminary evidence suggest that especially vigorous physical activity tends to be a better predictor of favorable sleep patterns [30, 68] in comparison to light and moderate physical activity [32, 34]. Second, the time of day in which physical activity occurred was not captured in the study, [34]. Third, the study does not allow any conclusion as to the causal direction of the observed pattern of associations. Fourth, any conclusions have to be made cautiously because the findings are based on a relatively small sample, and it is unclear whether this sample  is representative of  all adolescents in this age range since our data was collected in a sample of twins. Lastly, actigraphy does not capture physical activity associated with activities in which the wrist is stable – e.g., a participant riding a bicycle – and participants were instructed to take off the actigraph while swimming, which means that this form of physical activity could not be taken into account. A further limitation was that sleep on Friday night is not constrained in the morning and conversely sleep on Sunday night may be truncated due to the need to wake-up for school on Monday. However, because we are looking at within subjects effects (i.e., how physical activity during the day affects subsequent sleep) this was a compromise we had to make.

Despite these limitations, the major strength of this study was the long-term examination of the association between physical activity and sleep using different aspects of sleep (actigraphy, and subjective sleep quality) to obtain a comprehensive view of the topic. Furthermore, the objective assessment of sleep and activity over several months with the same device in the home environment enhances the reliability of our findings and allowed us to examine potential correlations between physical activity and sleep for both school and free days.

## Conclusions

In contrast to previous studies, we found a small and negative effect of physical activity on sleep with more steps on school and free days associated with later bedtimes and diminished sleep efficiency. We hypothesize that this may be due to the timing of physical activity (in the afternoon versus in the morning) and the impact of such activity on the circadian timing system. Despite this finding, the effect sizes in our study were small and our results should be interpreted with caution given the well-documented benefits of physical activity on health in adolescence. Our findings obtained through objective and long-term measurement of both sleep and steps emphasize the importance of the methodology and the separation of within subject from between subject features when examining the relationship between physical activity and sleep.

## Data Availability

The datasets used and/or analysed during the current study are available from the corresponding author on reasonable request.

## Abbreviations

ICC: Intra-class coefficient
TST: Total sleep time
SOL: Sleep onset latency
WASO: Wake after sleep onset
NOA: Number of awakenings
SE: Sleep efficiency
STEPS_M: Mean number of steps across all measurement days for each individual
STEPS_D: Baseline levels of steps calculated by subtracting the mean across all days for an individual by the number of steps per day
ADHD: Attention-deficit/hyperactivity disorder
